# LC-MS/MS-Based Determination of Ambroxol in Human Plasma and Cerebrospinal Fluid: Validation and Applicability in a Phase II Study on GBA-Associated Parkinson’s Disease Patients

**DOI:** 10.3390/ijms26136094

**Published:** 2025-06-25

**Authors:** Valentina Franco, Michela Palmisani, Fabiana Colucci, Rosa De Micco, Simone Aloisio, Federico Cazzaniga, Silvia Cerri, Francesca Crema, Francesca Dattrino, Barbara Garavaglia, Matteo Gastaldi, Pierfrancesco Mitrotti, Fabio Moda, Paola Rota, Rita Stiuso, Cristina Tassorelli, Roberto Eleopra, Alessandro Tessitore, Enza Maria Valente, Micol Avenali, Roberto Cilia

**Affiliations:** 1 Clinical and Experimental Pharmacology Unit, Department of Internal Medicine and Therapeutics, University of Pavia, 27100 Pavia, Italy; valentina.franco@unipv.it (V.F.); michela.palmisani01@universitadipavia.it (M.P.); francesca.crema@unipv.it (F.C.); francesca.dattrino01@universitadipavia.it (F.D.); 2 IRCCS Mondino Foundation, 27100 Pavia, Italy; silvia.cerri@mondino.it (S.C.); matteo.gastaldi@mondino.it (M.G.); pierfrancesco.mitrotti01@universitadipavia.it (P.M.); rita.stiuso@mondino.it (R.S.); cristina.tassorelli@unipv.it (C.T.); enzamaria.valente@unipv.it (E.M.V.); 3 Parkinson and Movement Disorders Unit, Department of Clinical Neurosciences, Fondazione IRCCS Istituto Neurologico Carlo Besta, 20133 Milano, Italy; fabiana.colucci@istituto-besta.it (F.C.); roberto.eleopra@istituto-besta.it (R.E.); roberto.cilia@istituto-besta.it (R.C.); 4 Department of Neuroscience and Rehabilitation, University of Ferrara, 44121 Ferrara, Italy; 5 Department of Advanced Medical and Surgical Sciences, University of Campania “Luigi Vanvitelli”, 80138 Naples, Italy; rosita.demicco@gmail.com (R.D.M.); aloisiosimone10@gmail.com (S.A.); alessandro.tessitore@unicampania.it (A.T.); 6 Laboratory of Clinical Pathology, Unit of Laboratory Medicine, Fondazione IRCCS Istituto Neurologico Carlo Besta, 20133 Milano, Italy; federico.cazzaniga@istituto-besta.it (F.C.); fabio.moda@istituto-besta.it (F.M.); 7 Unit of Medical Genetics and Neurogenetics, Fondazione IRCCS Istituto Neurologico Carlo Besta, 20133 Milano, Italy; barbara.garavaglia@istituto-besta.it; 8 Department of Brain and Behavioral Sciences, University of Pavia, 27100 Pavia, Italy; 9 Department of Medical Biotechnology and Translational Medicine, Università degli Studi di Milano, 20126 Milan, Italy; 10 Department of Biomedical, Surgical and Dental Sciences, University of Milan, 20133 Milan, Italy; paola.rota@unimi.it; 11 Institute for Molecular and Translational Cardiology (IMTC), San Donato Milanese, 20097 Milan, Italy; 12 Department of Molecular Medicine, University of Pavia, 27100 Pavia, Italy

**Keywords:** plasma, cerebrospinal fluid, LC-MS/MS, ambroxol, Parkinson’s Disease, glucocerebrosidase

## Abstract

Heterozygous mutations in the *GBA1* gene, encoding the enzyme glucocerebrosidase (GCase), are major risk factors for Parkinson’s Disease (PD). Ambroxol, a small chaperone originally used as a mucolytic agent, has been shown to cross the blood–brain barrier, enhance GCase activity, and reduce α-synuclein levels, making it a promising therapeutic candidate for disease-modifying effects in GBA1-associated PD (GBA1-PD). This study aimed to develop a method to quantify ambroxol levels in human plasma and cerebrospinal fluid (CSF) using liquid chromatography–tandem mass spectrometry (LC-MS/MS). Ambroxol was determined by online solid-phase extraction (SPE), coupled with LC-MS/MS, by gradient elution on a monolithic column. Detection employed a 3200 QTRAP tandem mass spectrometer in the positive electrospray ionization mode. Calibration curves exhibited linearity across the analyzed ranges in both plasma and CSF. The recovery rate ranged from 106.7% to 113.5% in plasma and from 99.0% to 103.0% in CSF. No significant matrix effect was observed. Intra-day and inter-day precisions were below 11.8% in both matrices, and accuracy ranged from 89.9% to 103.1% in plasma and from 96.3% to 107.8% in CSF. We evaluated and confirmed the stability of the analyte in plasma and CSF across various storage conditions. The method was successfully validated according to European Medicine Agency (EMA) guidelines and its applicability was confirmed in the context of a multicenter, randomized, double-blind, placebo-controlled, phase II study, designed to monitor the ambroxol levels in the plasma and CSF of GBA1-PD.

## 1. Introduction

Biallelic mutations in the *GBA1* gene cause a lysosomal lipid storage disorder, Gaucher’s Disease (GD) [1], while heterozygous mutations are known as the most common genetic risk factor for Parkinson’s Disease (PD) [2]. *GBA1* encodes glucocerebrosidase (GCase), a lysosomal enzyme that causes the degradation of the glycosphingolipid glucosylceramide into ceramide and glucose. *GBA1* mutations reduce GCase activity, alter the lipid composition of cellular membranes, and promote toxic α-synuclein aggregation and accumulation within lysosomes, ultimately inhibiting GCase function [3]. This vicious cycle is probably mediated by the intracellular elevation of glucosylceramide and glucosylsphingosine, the latter of which has not only been shown to cause the oligomerization of α-synuclein in vitro [4] but is also used as a clinically useful biomarker for GD severity. Although the exact pathogenic mechanism remains incompletely defined, experimental and clinical evidence suggests that reduced GCase activity leads to the impaired lysosomal degradation of α-synuclein, promoting its accumulation. In *GBA1* models and PD patients, an inverse correlation between GCase activity and α-synuclein levels is consistently observed, even in the absence of detectable lipid accumulation. This supports the hypothesis that GCase dysfunction contributes to α-synuclein aggregation via lysosomal impairment and secondary mechanisms such as endoplasmic reticulum stress and oxidative damage [3,5]. Consequently, current research is particularly focused on identifying therapeutic strategies to restore GCase’s enzymatic function. Ambroxol (trans-4-(2-amino-3,5-dibromobenzyl)-aminocyclo-hexanol), the pharmacologically active metabolite of the mucolytic agent bromhexine, has been recognized as a GCase chaperone: it increases GCase enzymatic activity and protein levels in cultured fibroblasts from PD patients, with or without *GBA1* mutations [6]. In PD subjects, ambroxol administered orally at a dose of 1260 mg/day reached the central nervous system (CNS) and increased GCase levels in the cerebrospinal fluid (CSF), where it also modified α-synuclein concentrations. Importantly, ambroxol therapy was safe and well tolerated, with no serious adverse events reported in this study [7]. Several liquid chromatography–tandem mass spectrometry (LC-MS/MS) techniques have been documented for the quantitation of ambroxol in human plasma [8,9,10,11,12,13,14,15,16,17,18,19,20,21]. Recently, Mullin et al. assessed its safety, tolerability, penetration into the CSF, and effectiveness in engaging the target of ambroxol therapy alongside GCase in patients with PD, both with and without *GBA1* mutations. The authors introduced an LC-MS/MS method for ambroxol assays in human serum and CSF, requiring 250 μL of matrix and 1 mL of acetonitrile (ACN) for protein precipitation [7]. The aim of the present study was twofold: first, we sought to develop and validate an improved LC-MS/MS approach, and second, we sought to assess its possible use in a phase II clinical trial to monitor ambroxol levels in human plasma and CSF using a simple protein precipitation with only 200 µL of ACN and online solid-phase extraction (SPE). Here, we report the features and performances of this method, which enabled the efficient extraction of analytes in only 50 µL of matrix and proved to be a robust and high-throughput analytical method. We also assessed the stability of the analyte in both plasma and CSF under different storage conditions and validated it on a clinical population in a multicenter, double-blind, placebo-controlled, phase II study performed to assess the role of ambroxol as a potential disease-modifying therapy for GBA1-PD [22]. This clinical context is important as ambroxol crosses the blood–brain barrier and has demonstrated the potential to enhance GCase activity and reduce α-synuclein pathology, supporting its investigation in disease-modifying therapy for GBA1-associated PD.

## 2. Results

### 2.1. HPLC-MS/MS Parameters Optimization

**Figure 1:**
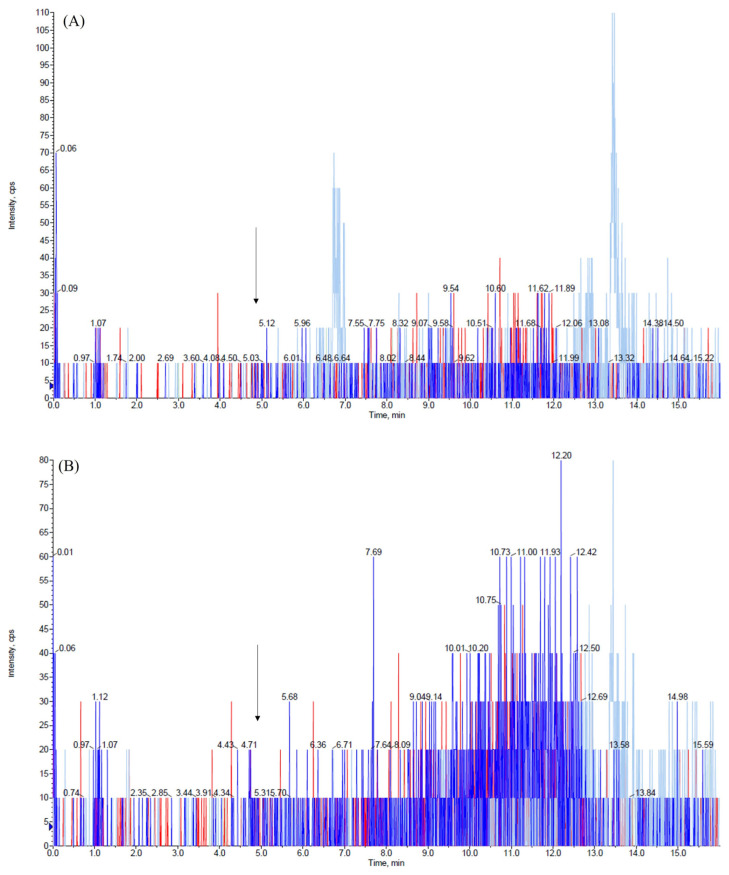
Representative chromatograms of blank (**A**) plasma and (**B**) cerebrospinal fluid samples. Arrows indicate peak of ambroxol and internal standard ambroxol-*d_5_*. Ambroxol (blu line); ambroxol-*d_5_* (red line).

**Figure 2:**
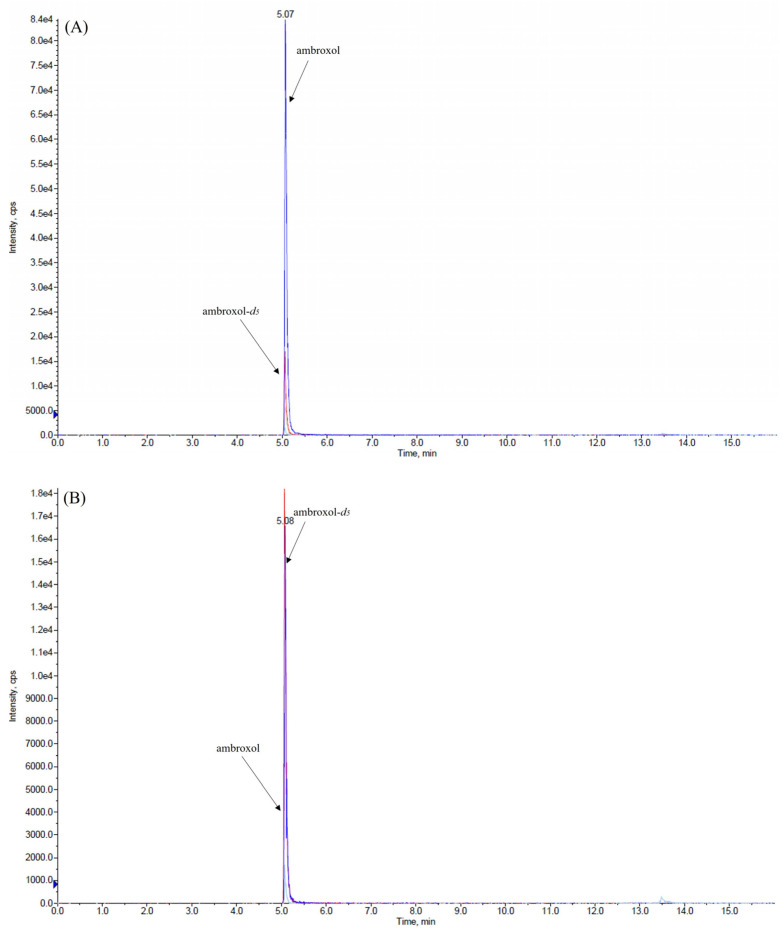
Representative chromatograms of (**A**) plasma and (**B**) cerebrospinal fluid samples spiked with medium-quality control (950 ng/mL in the plasma and 125 ng/mL in the cerebrospinal fluid). Ambroxol (blu line); ambroxol-*d_5_* (red line).

**Figure 3:**
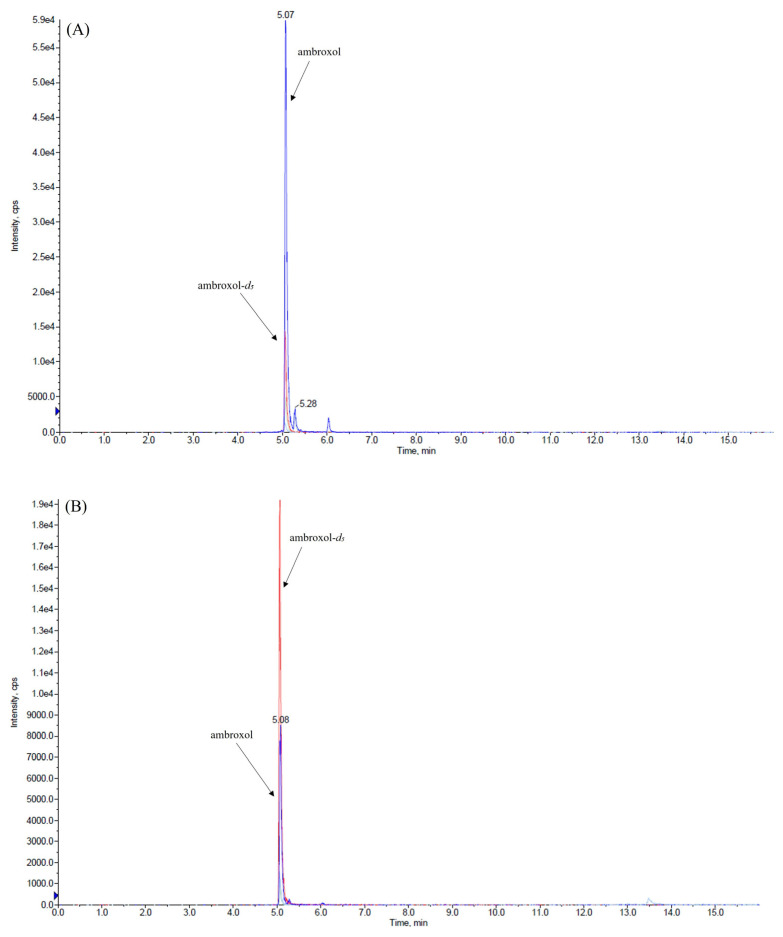
Representative chromatograms of (**A**) plasma and (**B**) cerebrospinal fluid samples of a patient taking ambroxol at a dosage of 1200 mg/day. The plasma and cerebrospinal fluid concentrations of ambroxol were 702 and 68 ng/mL, respectively. Ambroxol (blue line); ambroxol-*d_5_* (red line).

This validation was conducted using only 50 µL of both matrices while maintaining a high level of sensitivity and employing a very rapid and cost-effective method to prepare samples. We explored various combinations of mobile-phase compositions, finding that the inclusion of small amounts (0.2%, *v/v*) of formic acid in both aqueous and organic phases and the use of ammonium formate at different concentrations in the aqueous phase enhanced peak shape. The combination between the mobile-phase composition, composed of 2 mM ammonium formate water, and ACN (each containing 0.2% *v/v* formic acid) and the use of the monolithic C18 column (Onyx, 100 × 3 mm i.d., Phenomenex, Bologna, Italy) consistently yielded improved peak characteristics for the analytes. The retention time of ambroxol and internal standard (IS) ambroxol-*d_5_* was 5.1 min under the chromatographic conditions employed. We achieved the optimization of the method’s parameters, the fragmentation pattern of ambroxol, and the IS by infusing ambroxol and the IS into the mass spectrometer. The positive electrospray ionization (ESI) mode was chosen for the detection of analytes and two transitions were used for each analyte: one was used as a quantifier for both quantification and identification, while the other served as a qualifier to confirm the analyte’s identification, as described in the Materials and Methods section. Figure 1, Figure 2 and Figure 3 depict representative chromatograms of blank plasma and CSF samples, medium-quality control (QC) plasma and CSF samples, and plasma and CSF samples obtained from a patient treated with ambroxol 1200 mg/day.

### 2.2. Optimization of the Extraction Procedure

By employing a protein precipitation method with ACN in a 1:4 ratio of matrix to solvent, and coupling it with LC-MS/MS analysis using online SPE, effective sample purification has been achieved, along with significant time and cost savings compared to traditional liquid–liquid extraction and offline SPE methods. The decision to automate and integrate the online SPE streamlines the workflow, reduces manual handling, and minimizes solvent consumption and waste generation. This leads to lower operational and disposal costs. Furthermore, this approach mitigates matrix effects in both matrices, ensuring the integrity and optimal performance of the LC-MS/MS instrument, while also decreasing the frequency of maintenance and prolonging the instrument lifespan.

### 2.3. Assay Performance

**Figure 4:**
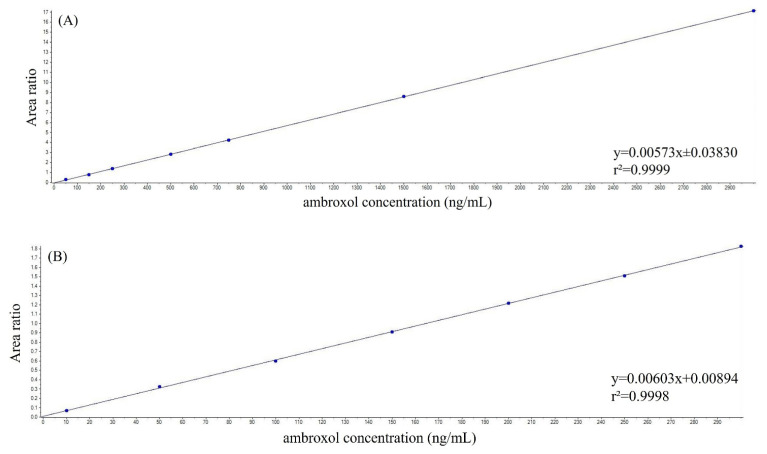
Representative calibration curves for ambroxol, measured in (**A**) plasma and (**B**) cerebrospinal fluid samples. The equations of the curves were y = 0.00573x ± 0.03830 and y = 0.00603x + 0.00894 in (**A**) plasma and (**B**) cerebrospinal fluid, respectively, while the r^2^ was greater than 0.999 in both matrices.

The calibration curves demonstrated linearity in both matrices. Specifically, the method was linear in the range of 50–3000 ng/mL for plasma and in the range of 10–300 ng/mL for CSF. These calibration curves were established over five separate days, yielding average slopes of 0.007 and 0.010 in plasma and CSF, respectively. Moreover, the coefficients of correlation were all higher than 0.999 in both matrices (Figure 4).

Precision, quantified in terms of coefficient of variation (CV%), and accuracy values, evaluated on the same day and on three different days, are presented in Table 1 and Table 2. Intra-day and inter-day precision remained below 11.8% and 11.3% in plasma and CSF, respectively. Intra-day and inter-day accuracy ranged from 89.9% to 103.1% and 96.3% to 107.8% in plasma and CSF, respectively. The lower limit of quantitation (LLOQ) was set at 50 ng/mL in plasma and 10 ng/mL in CSF, while the limit of detection (LOD) was established at 0.5 ng/mL for both matrices. The mean recoveries for ambroxol at low, medium, and high QC levels were 106.7%, 110.1%, and 113.5% in plasma, and 99%, 103%, and 100.6% in CSF. Additionally, the matrix effect was considered negligible for both matrices, with precision not exceeding 14.4% and 12.6% in plasma and CSF respectively, while accuracy ranging from 91.3% to 109.8% in plasma and from 86.4% to 102% in CSF. These results align with European Medicines Agency (EMA) guidelines on bioanalytical method validation [23]. Ambroxol in plasma and CSF samples were stable when kept at room temperature for up to 24 h, after three freeze–thaw cycles at −20 °C, after being in the autosampler for 48 h (Table 3 and Table 4), and when stored at −20 °C for at least 15 days (Table 5 and Table 6). Furthermore, the stability of ambroxol was confirmed in CSF samples stored at −20 °C and in plasma samples stored at −80 °C for 42 days, with no stability observed in plasma samples stored at −20 °C for the same duration (Table 7 and Table 8). Finally, the stability of ambroxol in working solutions was confirmed when stored at −20 °C for 7 days.

No interfering peaks were observed at the retention times of ambroxol and IS in plasma and CSF samples collected from six different healthy individuals. The carry-over of blank injections following the highest calibrator (3000 ng/mL in plasma sample and 300 ng/mL in CSF sample) was negligible for ambroxol and the IS thanks to the inclusion of a 4-min cleaning step with 100% mobile phase B in the chromatographic analytical run. The precision of the samples used for reinjection reproducibility was below 12.7% for both matrices, while the accuracy ranged between 98.8% and 103.6% for plasma samples and between 91.2% and 95.6% for CSF samples.

### 2.4. Analysis of Patient Samples

The mean ambroxol concentrations in plasma were 1165 ng/mL at V3 (n = 27) and 1387 ng/mL at V5 (n = 27), while it was not detectable at V6 (n = 50). The mean concentrations in the CSF at V5 was 118 ng/mL (n = 22). The accuracy values for the low-, medium-, and high-QC samples were 107.7%, 104.5%, and 99.9% in plasma, and 96.5%, 93.8%, and 99.5% in CSF, respectively. Precision values were 8.1%, 8.3%, and 7.9% for the low-, medium-, and high-QC samples in plasma, and 4.8%, 6.3%, and 7.1% for those in CSF, respectively.

## 3. Discussion

To date, several LC-MS/MS methodologies have been reported for the quantification of ambroxol in human plasma [8,9,10,11,12,13,14,15,16,17,18,19,20,21]. In a recent investigation by Mullin et al., an LC-MS/MS method was used to measure ambroxol levels in human serum and CSF, using 250 µL of biological fluid and 1 mL of ACN for protein precipitation [7].

In the present work, we developed and validated an enhanced LC-MS/MS method, designed to quantify ambroxol in human plasma and CSF, utilizing only 50 µL of matrix. The method targets the pharmacologically active compound ambroxol, as no active metabolites are known, and measures its levels to reflect relevant drug exposure in this clinical context. Furthermore, a comprehensive stability assessment of ambroxol was conducted in the tested matrices. Sample preparation was conducted using a rapid protein precipitation protocol, requiring only 200 µL of ACN, in combination with online SPE. This strategy ensures highly efficient analyte extraction from minimal sample volumes, supporting a robust and high-throughput analytical workflow. The integration of protein precipitation with online SPE markedly enhances sample processing efficiency compared to conventional extraction techniques, such as offline SPE or liquid–liquid extraction, by significantly reducing preparation time. Moreover, the elimination of an evaporation step further optimizes analytical throughput. The use of a monolithic column, coupled with mobile phases comprising 2 mM ammonium formate in water and ACN (each containing 0.2% *v/v* formic acid), enhances chromatographic resolution, ensuring precise and reproducible quantification. All evaluated parameters comply with current EMA regulatory guidelines [23].

In addition to its analytical performance, we assessed the stability of ambroxol under different storage conditions. Our findings indicate that ambroxol remains stable in CSF samples stored at −20 °C and in plasma samples stored at −80 °C for up to 42 days. Further assessments revealed that ambroxol remained stable in plasma and CSF samples left at room temperature for 24 h, subjected to three freeze–thaw cycles at −20 °C, stored in an autosampler for 48 h, or maintained at −20 °C for at least 15 days. This stability ensures reliable quantification, even in multicenter clinical trials where sample collection, transport, and storage may vary, thereby supporting the robustness of the assay and its practical applicability in real-world settings.

The improvements over existing protocols introduced by this method, particularly the significant reduction in sample volume and streamlined sample preparation, have important clinical implications. By requiring only 50 µL of plasma or CSF, this method facilitates pharmacokinetic monitoring in vulnerable patient populations, such as those with PD, where sample availability may be limited. The rapid and efficient workflow supports high-throughput analysis, enabling larger clinical studies or routine therapeutic drug monitoring.

Future research directions could include integrating pharmacokinetic data with pharmacodynamic and clinical outcomes to better understand the therapeutic role of ambroxol in GBA1-PD. Furthermore, adapting the method for other biological matrices could broaden its applicability.

The optimal performance of this LC-MS/MS method has been confirmed through its application in a multicenter, double-blind, placebo-controlled, phase II clinical trial, investigating the therapeutic potential of ambroxol as a disease-modifying agent for GBA1-PD. The high sensitivity, specificity, and analytical robustness of the assay enable precise and reliable pharmacokinetic assessments, thereby supporting its potential for expanded clinical applications [22].

## 4. Materials and Methods

### 4.1. Materials

**Figure 5:**
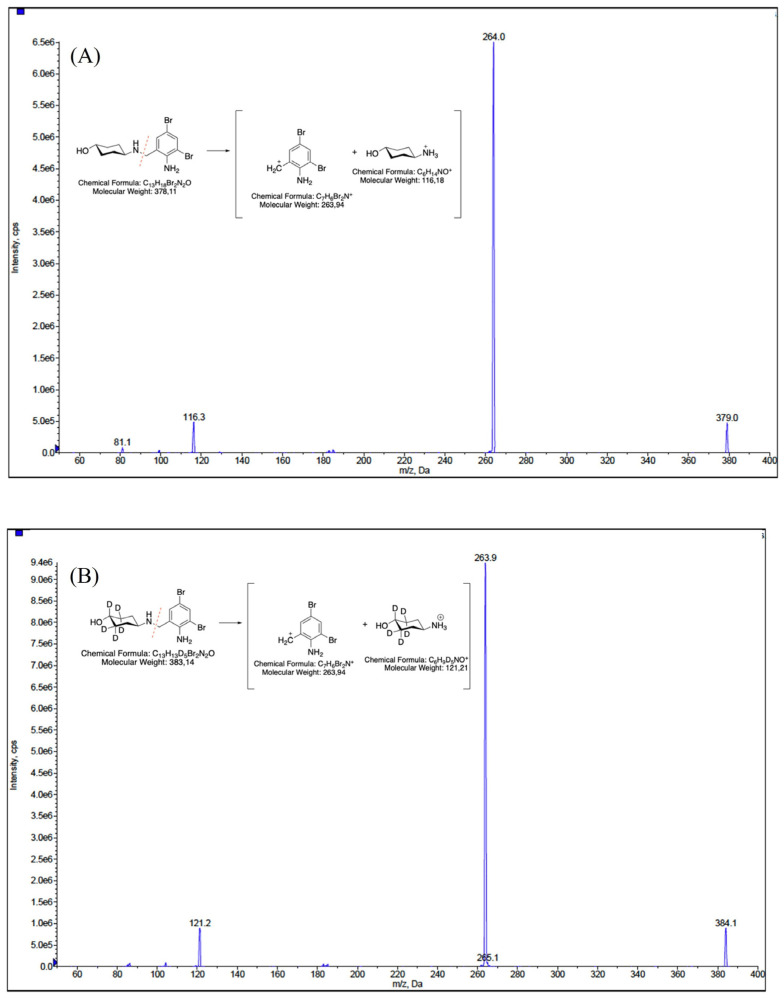
Tandem mass spectra and fragmentation pattern of (**A**) ambroxol and (**B**) internal standard ambroxol-*d_5_*.

Ambroxol (Figure 5) was purchased from Merck (Merck KGaA, Darmstadt, Germany). The IS ambroxol-*d_5_* (Figure 5) was obtained from Cayman Chemical (Cayman Chemical Company, Ann Arbor, MI, USA). Dimethyl sulfoxide (DMSO) (≥99.5%) was obtained from Sigma Aldrich (Sigma Aldrich, St. Louis, MO, USA). LC-MS grade ACN, 99% formic acid, and 99% ammonium formate for mobile-phase preparation were purchased from VWR (VWR International, Radnor, PA, USA). Ultrapure water was obtained from a Milli-Q Plus purification system (Millipore, Milan, Italy). Drug-free human plasma and CSF, used for QC sample and calibrator preparation, were obtained from healthy adult donors.

### 4.2. Plasma and CSF Sample Preparation

Plasma samples were obtained by centrifugation at 3800× *g* for 10 min at 4 °C, while CSF samples were obtained by centrifugation at 2200× *g* for 10 min at 4 °C. All samples were stored at −80 °C until analysis.

### 4.3. LC-MS/MS System

The HPLC-MS/MS setup comprised a 3200 QTRAP^®^ triple-quadrupole mass spectrometer coupled with an integrated ExionLC 100 HPLC system featuring a quaternary low-pressure mixing pump, a column oven, an autosampler, a degasser, and a controller (Applied Biosystems Sciex, Darmstadt, Germany). Data acquisition and processing were conducted using Analyst software version 1.6.3 and MultiQuant version 3.0.2 (Applied Biosystems Sciex, Darmstadt, Germany).

### 4.4. HPLC and Mass Spectrometer Conditions

Perfusion column cleanup and enrichment were conducted online using a POROS R1 column (2.1 × 30 mm i.d., 20 μm, Thermo Fisher Scientific, Waltham, MA, USA). The analytes were separated on a monolithic C18 column (Onyx, 100 × 3 mm i.d., Phenomenex, Bologna, Italy), maintained at 25 °C. Mobile phase A consisted of a solution of water with 2 mM ammonium formate and 0.2% formic acid and mobile phase B consisted of ACN with 0.2% formic acid. The total runtime was 16 min. In the online SPE process, 10 µL of sample was introduced into a POROS perfusion column. Flow path switching between the SPE and LC–MS/MS modules was executed using a 10-port valve: after 0.1 min, there was a switch of the 10-port switching valve from the loading position, where the column was inserted, to the injection position. During this step, the flow rate utilized was 1 mL/min with a mixture comprising 98% mobile phase A and 2% mobile phase B for 1 min. Analytic separation started when the valve switched from the injection position to the load position. The separation was achieved through a gradient starting at 85% mobile phase A and 15% mobile phase B, which was then ramped to 15% mobile phase A and 85% mobile phase B over 9 min at a flow rate of 0.5 mL/min. Additionally, a cleaning step was performed, involving a 4-min flush of 100% mobile phase B at a flow rate of 0.5 mL/min. Following this, the columns were reconditioned for 2 min under the initial conditions, with a flow rate of 0.5 mL/min. ESI was utilized in positive ion mode for MS detection, employing multiple reaction monitoring (MRM). For ambroxol, the precursor fragment ions monitored were 379 *m*/*z* → 264 *m*/*z* (quantifier ion) and 116.3 *m*/*z* (qualifier ion), while for the IS, the ions monitored were 384.1 *m*/*z* → 263.9 *m*/*z* (quantifier ion) and 121.2 *m*/*z* (qualifier ion). The instrument’s optimized parameters were configured as follows: an ion spray voltage of 5500 V, a curtain gas pressure at 25 psi, an ion source gas 1 at 50 psi, an ion source gas 2 at 60 psi, a temperature set to 600 °C, a declustering potential of 60 V, an entrance potential of 10 V, a collision energy of 30 V, a collision cell exit potential of 4 V, and a dwell time of 100 msec for all transitions. The nitrogen generator model Genius ABN2ZA (PEAK Scientific, Inchinnan, Scotland) was used as the gas generation system for nitrogen flow.

### 4.5. Calibration Standards, Quality Control and Unknown Samples

Ambroxol (1 mg/mL) and IS (1 mg/mL) were initially dissolved in DMSO (≥99.5%) and ACN, respectively, to prepare stock solutions. These solutions were diluted with ACN to obtain working standard solutions at concentrations of 50, 150, 250, 500, 750, 1500, and 3000 ng/mL in plasma and 10, 50, 100, 150, 200, 250, and 300 ng/mL in CSF. The working IS solution was prepared at a concentration of 140 ng/mL. Freshly prepared calibrators were made for each analytical run by combining 50 µL of blank matrix, 50 µL of the working IS solution, 50 µL of working solutions containing ambroxol, and 100 µL of ACN with 0.2% formic acid. QC samples containing ambroxol at concentrations of 100 ng/mL (low), 950 ng/mL (medium), and 2500 ng/mL (high) in plasma and at concentrations of 25 ng/mL (low), 125 ng/mL (medium), and 275 ng/mL (high) in CSF were prepared from separate stock solutions. Unknown samples were prepared by mixing 50 µL of plasma or CSF with 50 µL of the working IS solution, 50 µL of ACN, and 100 µL of ACN with 0.2% formic acid. All the obtained samples were vortexed and centrifuged at 4 °C for 10 min at 17,000× *g* and 10 µL of the supernatant was injected in the LC-MS/MS system.

### 4.6. Method Validation

After method optimization, the validation process was performed according to a laboratory protocol, which was guided by EMA guidelines for bioanalytical method validation [23]. The validation encompassed parameters including precision, accuracy, the LLOQ, LOD, linearity, and analyte stability under different conditions, recovery, matrix effect, selectivity, specificity, carry-over, and reinjection reproducibility. The LLOQ was defined as the minimum concentration of the analyte that can be measured reliably and precisely, while the LOD was defined as the concentration level of the calibrator, where the signal is at least three times greater than the background noise. Precision and accuracy were assessed on the same day (n = 5) and over three days (n = 15) at LLOQ and low, medium, and high QC concentrations. Precision was quantified in terms of CV% with the assay evaluated as satisfactory if the CV remained below 15% at every concentration. Accuracy was evaluated by comparing the mean values of the LLOQ and QC assay outcomes against the anticipated concentrations, with results expected to fall within a 15% margin of the nominal values. For LLOQ, the intra-batch and inter-batch CV% should be <20%, while accuracy should be within ±20%. Linearity was determined across the seven concentrations used for calibration curves and all data were subjected to linear regression analysis. These curves were generated by plotting the ratios of ambroxol/IS peak areas against the nominal concentrations of ambroxol in the calibrators. The correlation coefficient was used as a measure of the fit quality. For the assessment of ambroxol stability in plasma and CSF, we used low and high QC samples (n = 4). We compared assay values derived from newly prepared extracts with those from extracts preserved at equivalent concentration levels under different conditions: 24 h at room temperature, storage for 15 days at −20 °C and for 42 days at −20 °C and −80 °C, and exposure to three freeze–thaw cycles. Additionally, the stability of the extracts was assessed by keeping the extracted samples in the autosampler at room temperature for 48 h. The stability of working solutions was tested after seven days at −20 °C. The recovery assessment was carried out utilizing low, medium, and high QC concentrations. This involved comparing the peak area of ambroxol obtained from plasma and CSF samples (n = 5) to the peak area of ambroxol obtained from samples consisting solely of water and solvent at equivalent concentrations (n = 5). The evaluation of the matrix effect was determined using matrices obtained from six distinct individuals. The precision and accuracy of each matrix were evaluated through the analysis of three replicates of both low and high QC samples. Selectivity and specificity were evaluated by analyzing blank plasma and CSF samples obtained from six different individuals. Carry-over was assessed by injecting blank plasma and CSF samples following the highest calibrator. The peak areas of ambroxol and IS could not exceed 20% and 5%, respectively, of their responses at the LLOQ. Additionally, reinjection reproducibility was assessed by reinjecting a batch, followed by the verification of precision and accuracy across low, medium, and high QC concentrations (n = 5).

### 4.7. Statistical Analysis

Statistical analysis of the results was performed by using GraphPad Prism version 8.2.1 (GraphPad Software Inc., San Diego, CA, USA). To assess stability parameters, we employed repeated measures analysis of variance (ANOVA) with Bonferroni’s multiple comparisons test as the post hoc test and the unpaired *t*-test. A *p*-value < 0.05 was considered statistically significant. All concentrations were expressed as ng/mL and data presented as mean ± standard deviation (SD).

### 4.8. Clinical Applicability in a Phase II Study

This study aimed to develop and validate a reliable and sensitive LC-MS/MS method for the quantification of ambroxol in human plasma and CSF using a fully automated online SPE procedure. The validated method was then applied in a multicenter, double-blind, placebo-controlled, phase II clinical trial (AMBITIOUS), designed to assess the role of ambroxol as a potential disease-modifying therapy in patients with GBA1-PD, the most frequent genetic form of PD, associated with faster cognitive decline and reduced survival [22]. In this phase II trial, participants were randomized in a 1:1 ratio to receive either oral ambroxol at a daily dose of 1200 mg (galenic formulation) or placebo for a total treatment duration of 52 weeks. Clinical assessments were conducted at baseline and at weeks 12, 26, 38, 52, and 78. The primary outcome was the change in Montreal Cognitive Assessment scores and the frequency of mild cognitive impairment and dementia between baseline and week 52. Secondary outcomes included changes in validated scales and questionnaires evaluating motor and non-motor symptoms, as well as surrogate markers of disease progression such as neuroimaging features and CSF neurodegeneration biomarkers. GCase enzymatic activity, ambroxol concentration, and α-synuclein levels were measured in both plasma and CSF. In this context, the present validated analytical method was crucial for the accurate and reproducible quantification of ambroxol at the specified time points (plasma: weeks 26 and 52; CSF: week 52), allowing the performance of pharmacokinetic and pharmacodynamic analyses aligned with the trial endpoints [22].

Inclusion criteria were (1) an age between 21 to 80 years old; (2) PD diagnosis based on the International Parkinson and Movement Disorders Society (MDS) criteria [24]; (3) a minimum disease duration of 5 years from the onset of motor symptoms; (4) a Hoehn and Yahr stage of ≤4 during the ON phase; (5) the absence of contraindications for study procedures; and (6) a commitment to and compliance with contraception. Exclusion criteria were (1) a diagnosis of atypical parkinsonism; (2) a diagnosis of Parkinson’s Disease Dementia according to the MDS criteria [25,26]; (3) the presence of deep brain stimulation; (4) the presence of relevant or unstable comorbidities; (5) the diagnosis of bronchial asthma; (6) medical conditions preventing safe lumbar puncture execution (e.g., treatment with anticoagulants; significant bleeding disorders such as bleeding diathesis, coagulopathy, or thrombocytopenia; severe abnormalities, malformations of the lower spine, or other spinal pathologies; hypersensitivity to lidocaine); or (7) known allergy to ambroxol or the excipients in the formulation.

## 5. Conclusions

We successfully validated an improved bioanalytical assay to quantify ambroxol in human plasma and CSF by using an automated SPE-LC-MS/MS system. Our straightforward approach presents the advantage of rapid sample preparation with minimal plasma and CSF sample requirements (50 µL). The integration of online SPE is essential for reducing manual sample preparation, effectively eliminating most matrix interferences, and minimizing background noise, leading to the sensitivity required for clinical pharmacokinetic studies.

Furthermore, ambroxol stability was tested and confirmed under different storage conditions. Under the optimized conditions, the method was successfully validated following EMA guidelines and its clinical applicability was confirmed in a multicenter, randomized, double-blind, placebo-controlled, phase II study to monitor ambroxol levels in the plasma and CSF of GBA1-PD subjects.

## Figures and Tables

**Table 1:** Intra-day and inter-day precision (CV%) and accuracy (%) of ambroxol assessed in plasma samples at four levels of concentration: LLOQ, low QC, medium QC, and high QC.

Parameter	LLOQ 50 ng/mL	Low QC 100 ng/mL	Medium QC 950 ng/mL	High QC 2500 ng/mL
Intra-day precision and accuracy (n = 5)				
Measured concentration (ng/mL)	48.3 ± 5.4	96.5 ± 9.0	915.0 ± 107.8	2246.4 ± 106.5
Precision (CV%)	11.2	9.4	11.8	4.7
Accuracy (%)	96.6	96.5	96.3	89.9
Inter-day precision and accuracy (n = 15)				
Measured concentration (ng/mL)	51.5 ± 4.5	100.6 ± 7.4	943.4 ± 72.5	2363.9 ± 128.6
Precision (CV%)	8.8	7.4	7.7	5.4
Accuracy (%)	103.1	100.6	99.3	94.6

**Table 2:** Intra-day and inter-day precision (CV%) and accuracy (%) of ambroxol assessed in CSF samples at four levels of concentration: LLOQ, low QC, medium QC and high QC.

Parameter	LLOQ 10 ng/mL	Low QC 25 ng/mL	Medium QC 125 ng/mL	High QC 275 ng/mL
Intra-day precision and accuracy (n = 5)				
Measured concentration (ng/mL)	10.8 ± 1.1	25.3 ± 1.1	123.0 ± 13.9	265.0 ± 26.4
Precision (CV%)	10.1	4.2	11.3	10.0
Accuracy (%)	107.8	101.4	98.4	96.3
Inter-day precision and accuracy (n = 15)				
Measured concentration (ng/mL)	9.7 ± 1.1	24.4 ± 1.3	129.2 ± 9.4	278.4 ± 19.0
Precision (CV%)	11.0	5.3	7.3	6.8
Accuracy (%)	97.1	97.5	103.3	101.2

**Table 3:** Ambroxol stability in plasma samples at room temperature for up to 24 h, after three freeze–thaw cycles at −20 °C and after being in the autosampler for 48 h at low- and high-QC concentrations.

Stability Condition	Low QC 100 ng/mL	High QC 2500 ng/mL
Fresh samples (ng/mL)	102.9 ± 6.5	2452.0 ± 70.3
Precision (CV%)	6.4	2.9
Accuracy (%)	102.9	98.1
24 h at room temperature (ng/mL)	100.7 ± 2.7	2403.8 ± 84.2
Precision (CV%)	2.7	3.5
Accuracy (%)	100.7	96.2
Three freeze–thaw cycles (ng/mL)	99.3 ± 6.5	2407.9 ± 44.5
Precision (CV%)	6.6	1.8
Accuracy (%)	99.3	96.3
48 h at room temperature in autosampler	102.1 ± 6.5	2471.3 ± 75.6
Precision (CV%)	6.4	3.1
Accuracy (%)	102.1	98.9

**Table 4:** Ambroxol stability in CSF samples at room temperature for up to 24 h, after three freeze–thaw cycles at −20 °C and after being in the autosampler for 48 h at low- and high-QC concentrations.

Stability Condition	Low QC 25 ng/mL	High QC 275 ng/mL
Fresh samples (ng/mL)	26.6 ± 1.1	268.6 ± 12.0
Precision (CV%)	4.3	4.5
Accuracy (%)	106.4	97.7
24 h at room temperature (ng/mL)	26.0 ± 1.3	254.8 ± 5.6
Precision (CV%)	4.8	2.2
Accuracy (%)	104.2	92.7
Three freeze–thaw cycles (ng/mL)	25.3 ± 0.3	270.1 ± 7.6
Precision (CV%)	1.3	2.8
Accuracy (%)	101.3	98.2
48 h at room temperature in autosampler	27.1 ± 0.5	270.3 ± 4.9
Precision (CV%)	1.7	1.8
Accuracy (%)	108.3	98.3

**Table 5:** Ambroxol stability in plasma samples at −20 °C after 15 days at low- and high-QC concentrations.

Stability Condition	Low QC 100 ng/mL	High QC 2500 ng/mL
Fresh samples (ng/mL)	110.9 ± 4.2	2341.8 ± 31.9
Precision (CV%)	3.8	1.4
Accuracy (%)	110.9	93.7
15 days at −20 °C (ng/mL)	109.5 ± 4.3	2206.7 ± 112.6
Precision (CV%)	4.0	5.1
Accuracy (%)	109.5	88.3

**Table 6:** Ambroxol stability in CSF samples at −20 °C after 15 days at low- and high-QC concentrations.

Stability Condition	Low QC 25 ng/mL	High QC 275 ng/mL
Fresh samples (ng/mL)	23.8 ± 1.5	262.6 ± 22.9
Precision (CV%)	6.2	8.7
Accuracy (%)	95.3	95.5
15 days at −20 °C (ng/mL)	22.5 ± 1.2	246.2 ± 6.7
Precision (CV%)	5.4	2.7
Accuracy (%)	89.9	89.5

**Table 7:** Ambroxol stability in CSF samples at −20 °C after 42 days at low- and high-QC concentrations.

Stability Condition	Low QC 25 ng/mL	High QC 275 ng/mL
Fresh samples (ng/mL)	23.9 ± 1.7	254.9 ± 4.4
Precision (CV%)	7.1	1.7
Accuracy (%)	95.4	92.7
42 days at −20 °C (ng/mL)	22.2 ± 0.6	244.7 ± 6.7
Precision (CV%)	2.9	2.7
Accuracy (%)	88.8	89.0

**Table 8:** Ambroxol stability in plasma samples at −80 °C after 42 days at low- and high-QC concentrations.

Stability Condition	Low QC 100 ng/mL	High QC 2500 ng/mL
Fresh samples (ng/mL)	96.6 ± 2.5	2393.4 ± 60.3
Precision (CV%)	2.6	2.5
Accuracy (%)	96.6	95.7
42 days at −80 °C (ng/mL)	93.5 ± 5.4	2354.5 ± 34.9
Precision (CV%)	5.8	1.5
Accuracy (%)	93.5	94.2

## Data Availability

The data presented in this study are available on request from the corresponding author.

## Abbreviations

The following abbreviations are used in this manuscript:

ACN	Acetonitrile
CNS	Central nervous system
CSF	Cerebrospinal fluid
CV%	Coefficient of variation
DMSO	Dimethyl sulfoxide
EMA	European Medicine Agency
ESI	Electrospray ionization
GD	Gaucher’s Disease
GCase	Glucocerebrosidase
IS	Internal standard
LC-MS/MS	Liquid chromatography–tandem mass spectrometry
LOD	Limit of detection
LLOQ	Lower limit of quantitation
MDS	Parkinson and Movement Disorders Society
MRM	Multiple reaction monitoring
PD	Parkinson’s Disease
QC	Quality control
SPE	Solid-phase extraction
SD	Standard deviation
